# Modeling Depolarization Delay, Sodium Currents, and Electrical Potentials in Cardiac Transverse Tubules

**DOI:** 10.3389/fphys.2019.01487

**Published:** 2019-12-10

**Authors:** Sarah Helena Vermij, Hugues Abriel, Jan Pavel Kucera

**Affiliations:** ^1^Institute of Biochemistry and Molecular Medicine, University of Bern, Bern, Switzerland; ^2^Department of Physiology, University of Bern, Bern, Switzerland

**Keywords:** cardiomyocytes, transverse tubules, action potential, voltage-gated sodium channels, voltage-gated calcium channels

## Abstract

T-tubules are invaginations of the lateral membrane of striated muscle cells that provide a large surface for ion channels and signaling proteins, thereby supporting excitation–contraction coupling. T-tubules are often remodeled in heart failure. To better understand the electrical behavior of T-tubules of cardiac cells in health and disease, this study addresses two largely unanswered questions regarding their electrical properties: (1) the delay of T-tubular membrane depolarization and (2) the effects of T-tubular sodium current on T-tubular potentials. Here, we present an elementary computational model to determine the delay in depolarization of deep T-tubular membrane segments as the narrow T-tubular lumen provides resistance against the extracellular current. We compare healthy tubules to tubules with constrictions and diseased tubules from mouse and human, and conclude that constrictions greatly delay T-tubular depolarization, while diseased T-tubules depolarize faster than healthy ones due to tubule widening. Increasing the tubule length non-linearly delays the depolarization. We moreover model the effect of T-tubular sodium current on intraluminal T-tubular potentials. We observe that extracellular potentials become negative during the sodium current transient (up to −40 mV in constricted T-tubules), which feedbacks on sodium channel function (self-attenuation) in a manner resembling ephaptic effects that have been described for intercalated discs where opposing membranes are very close together. The intraluminal potential and sodium current self-attenuation however greatly depend on sodium current conductance. These results show that (1) the changes in passive electrical properties of remodeled T-tubules cannot explain the excitation–contraction coupling defects in diseased cells; and (2) the sodium current may modulate intraluminal potentials. Such extracellular potentials might also affect excitation–contraction coupling and macroscopic conduction.

## Introduction

Transverse (T-)tubules are deep invaginations of the lateral membrane of skeletal and cardiac muscle cells. In mammalian ventricular cardiomyocytes, T-tubules form a complex network throughout the cell, especially in species with high heart rates such as mice (Pinali et al., 2013; Jayasinghe et al., 2015), and carry many ion channels and regulatory proteins [reviewed in Bers (2002), Hong and Shaw (2017), and Bhogal et al. (2018)]. Consequently, T-tubules function as a platform for excitation–contraction coupling and signaling, which is essential for the initiation and regulation of muscle contraction (Hong and Shaw, 2017). Importantly, T-tubular remodeling has been reported for several cardiac diseases (Crocini et al., 2017; Crossman et al., 2017a). In particular, T-tubules widen (Wagner et al., 2012; Pinali et al., 2017; Seidel et al., 2017). Understanding the electrical properties of T-tubules in health and disease is therefore paramount to understanding cardiac physiology and pathophysiology. Several questions regarding the electrical properties of T-tubules however remain largely open (Vermij et al., 2019).

A first question that has hardly been addressed concerns the delay after which deep segments of T-tubules depolarize. Based on measurements of dextran diffusion out of T-tubules and corresponding modeling of this diffusion process, Uchida and Lopatin (2018) recently calculated that T-tubular constrictions and dilations increase the time constant of membrane depolarization from ∼10 to ∼100 μs, but they did not assess in their experiments the delay of membrane depolarization of deep T-tubular membrane. In this work, we present an *in silico* model of a simple T-tubule, an overall constricted tubule, and a tubule with successive constrictions. We quantify the depolarization delay of deep T-tubular segments compared to cell surface, and show that the threshold of voltage-gated channels deep in the cell will be reached slightly later than near the surface. We also assess the effects of variations in length, membrane capacitance, extracellular resistivity, and resting membrane conductance on T-tubular depolarization, as these parameters may vary in health and disease (Fu and Hong, 2016; Crossman et al., 2017b; Hong and Shaw, 2017).

A second question concerns the role played by T-tubular sodium current. Although the existence of a T-tubular pool of sodium channels is still under debate (Rougier et al., 2019), several studies have suggested that sodium channels are present in T-tubules and that the T-tubular sodium current may be substantial (Maier et al., 2004; Mohler et al., 2004; Brette and Orchard, 2006; Westenbroek et al., 2013; Koleske et al., 2018; Ponce-Balbuena et al., 2018). To date, the effects of a T-tubular sodium current and the interactions between the sodium current and the extracellular potentials have scarcely been investigated. The effects of a large T-tubular sodium current have been already simulated using an elaborate 3D model of T-tubules without branches, constrictions, and dilations (Hatano et al., 2015). With a sodium current density of 30 mS/μF, the extracellular potential was slightly negative (−1 mV), and sodium current was 8% smaller than at the cell surface. The authors did however not investigate this phenomenon or discuss its physiological importance in further detail. Therefore, in the present work, we extended our model with a T-tubular sodium current. We explore extracellular potentials in our T-tubular model with and without constrictions, investigate the biophysical properties and magnitude of the sodium current throughout the T-tubule, and discuss the physiological implications. Lastly, we compare the effects of two sodium current models (Luo and Rudy, 1991; ten Tusscher et al., 2004) and different sodium current conductances on extracellular potentials and sodium current biophysical properties.

Taken together, this work contributes to the fundamental understanding of passive and active electrical behavior of cardiac T-tubules in health and disease.

## Materials and Methods

**FIGURE 1:**
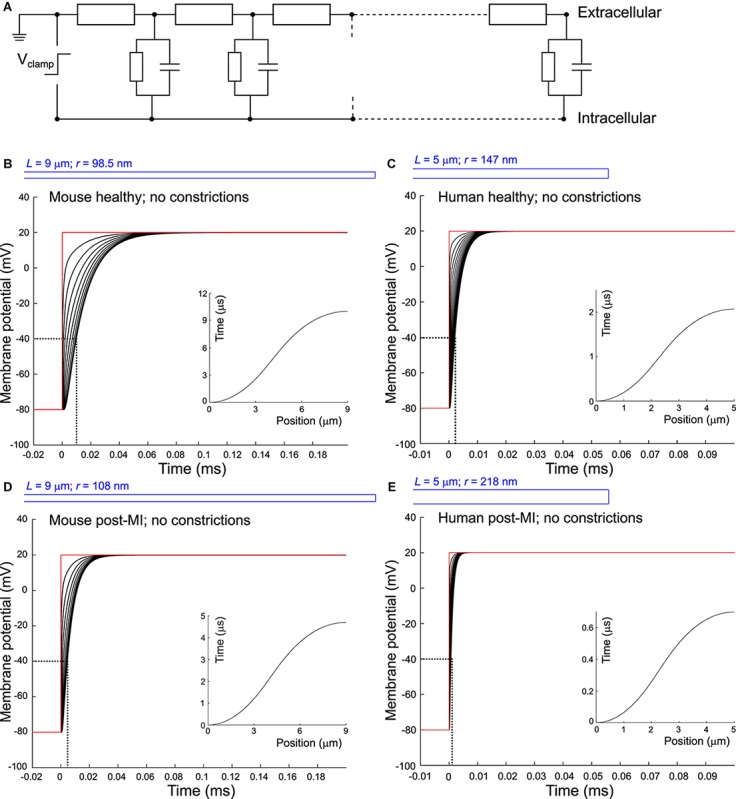
Delay of membrane depolarization in murine and human T-tubules. **(A)** Schematic representation of the model. T-tubule is subdivided in segments consisting of intraluminal resistance, membrane capacitance, and membrane resistance. Cytosolic resistance is assumed negligible and T-tubular mouth is assumed perfectly clamped. Panels **(B–E)** depict simulations of different T-tubules: healthy mouse **(B)**, healthy human **(C)**, mouse post-myocardial infarction (MI) **(D)**, and human post-myocardial infarction **(E)**. Dotted lines indicate the opening threshold for voltage-gated calcium channels (around –40 mV). Insets in panels **(B–E)** represent the time of depolarization to –40 mV vs. position along the T-tubule. T-tubular geometries are schematically depicted in blue and detailed in Table 1. Membrane potentials of every tenth **(B,D)** and second **(C,E)** node are depicted. Note the different axes in panels **(C)** and **(E)**.

Our model approximated one T-tubule as a cylinder of length *L* that was divided in *N* segments of length d*x* (*N* = *L*/d*x*; Figure 1A). For mouse T-tubules, d*x* was 90 nm; for human T-tubules, d*x* was 100 nm. Nominal values of *L* are given in Table 1. T-tubular radius, length, extracellular resistivity, membrane capacitance, and membrane conductance were set as in Table 1. In simulations aimed at studying the effects of tubular constrictions, the radius *r* of the tubule was varied along the tubule to simulate predefined constriction patterns. Individual segments were then represented as truncated cones. The periodicity of constrictions is based on Savio-Galimberti et al. (2008) and Uchida and Lopatin (2018).

**TABLE 1:** Parameters for T-tubular model of healthy and post-myocardial (MI) T-tubules of human and mouse.

**Species**	***r* (nm)**	***L* (μm)**	**ρ_e_ (Ω cm)**	***C*_m_ (μF/cm^2^)**	***g*_m_ (mS/cm^2^)**	**Nodes**
Healthy mouse	98.5_(__1__)_	9_(__2__)_	100	2	0.143	101
Constrictions	9.85_(__1__)_	0.45^∗^/9^∗∗^	100	2	0.143	5^∗^/101^∗∗^
Post-MI mouse	108_(__1__)_	9_(__2__)_	100	1	0.143	101
Healthy human	147_(__3__)_	5_(__4__)_	100	2	0.143	21
Post-MI human	218_(__3__)_	5_(__4__)_	100	1	0.143	21

*Radius (*r*) and length (*L*) are based on previously published values. Constrictions of healthy mouse T-tubule are also specified: ^∗^, parameters of each of the constrictions from the five-constrictions model; ^∗∗^, parameters of the overall constricted model. Numbers between brackets correspond to the following publications: (1), Wagner et al., 2012; (2), Hong and Shaw, 2017; (3), Crossman et al., 2017b; and (4), Cannell et al., 2006; Jayasinghe et al., 2015. The resistivity of the extracellular space (ρ_*e*_) is set at 100 Ω cm. Conductance of resting membrane (*g*_*m*_) is set at 0.143 mS/cm^2^ (Uchida and Lopatin, 2018), which is attributable to a small *I*_*K1*_ and leak currents. Capacitance (*C*_*m*_) was set at 2 μF/cm^2^ in healthy cells to simulate microfolds, and at 1 μF/cm^2^ to simulate the loss of microfolds in disease (Page, 1978; Hong et al., 2014).*

T-tubules are typically tortuous. Unless the tubules are extremely convolved, i.e., as long as the radius of the tubule remains much smaller than the curvature of the tortuosity, tortuosity can be modeled as an increased length. Moreover, glycosaminoglycans, or collagen as observed in T-tubules from diseased hearts (Crossman et al., 2017b) may pose an additional resistance to the intratubular current. Thus, in selected simulations, *L* and extracellular resistivity (ρ_*e*_) were systematically varied. In this T-tubular model, sodium current in the different nodes was modeled according to Luo and Rudy (1991) with modifications by Livshitz and Rudy (2009). In this model, sodium current density *I*_Na_ (normalized to membrane capacitance) is given by

(1) I=NagmNah3j(V-mE)Na

where *g*_Na_ is the maximal conductance of the sodium current [set unless specified otherwise to 23 mS/μF (Luo and Rudy, 1991)], *V*_m_ is the membrane potential, *E*_Na_ is the Nernst potential of sodium (set to +55 mV), *m* is the activation gating variable, and *h* and *j* are inactivation gating variables. Note that a *g*_Na_ of 23 mS/μF was determined in whole-cell recordings in chick embryonic heart cells (Ebihara and Johnson, 1980; Ebihara et al., 1980). This is in the same order of magnitude as the *g*_Na_ of 19.4 mS/μF determined in rat ventricular cardiomyocytes (Brette and Orchard, 2006). However, this value is probably too high for the T-tubules, as the intercalated discs carry a significant proportion of the whole-cell sodium current (Lin et al., 2011; Shy et al., 2014); therefore, *g*_Na_ values are also systematically varied in selected simulations.

The sodium current gating variables were governed by differential equations of the form

(2) $$\frac{\text{d}\,y}{\text{d}\,t}=\frac{y_{\infty }-y}{{\text{τ}}_{\text{y}}}\;y\in \left\{m,h,j\right\}$$

with *y*_∞_ and τ_y_ being functions of voltage given explicitly in Luo and Rudy (1991) and Livshitz and Rudy (2009). To test how results are influenced by the choice of the sodium current model, selected simulations were run using the sodium current model of ten Tusscher et al. (2004), which follows the same formulation with the same gates *m*, *h*, and *j* but different functions *y*_∞_ and τ_y_.

A voltage pulse from −85 to −20 mV was applied at the mouth of the tubule (−80 to +20 mV for simulations without sodium current). This situation mimics a cell that is perfectly voltage clamped at the level of its bulk membrane. The value of −20 mV was chosen to elicit a large sodium current. Calcium channels were not included in the model as these channels open only when the majority of sodium channels have already inactivated.

The intracellular potential was considered spatially uniform, set to the value given by the voltage clamp protocol. This assumption is justified based on the following estimation of the potential generated by the current emanating from a long cylinder in a conductive medium. We consider a cylinder of radius *r* in an extended medium of resistivity ρ and a surface producing current with a density *J* (for the case of the sodium current, *J* = *c*_m_⋅*I*_Na_, where *c*_m_ is the membrane capacitance per unit area in μF/cm^2^ and *I*_Na_ is the sodium current density in absolute value, in μA/μF). By applying the principle of charge conservation and Gauss’ theorem, and taking advantage of the radial symmetry of the problem (Plonsey and Barr, 2007), the current density *j*(*x*) at a distance *x* from the cylinder center can be calculated as *j*(*x*) = *Jr*/*x*. The electric field *E* at distance *x* can then be calculated from Ohm’s law as *E*(*x*) = ρ⋅*j*(*x*), and the potential difference φ(*d*)–φ(*r*) between a point at distance *d* from the cylinder center and a point at the cylinder surface can be obtained by integration of *E* as

$$\text{ϕ}\,\left(d\right)-\text{ϕ}\,\left(r\right)=\int_{r}^{d}E\,\text{d}\,x=c_{\text{m}}\,I_{\text{Na}}\,\text{ρ}\,r\,\int_{r}^{d}\frac{1}{x}\,\text{d}\,x$$

(3) $$=c_{\text{m}}\,I_{\text{Na}}\,\text{ρ}\,r\cdot \text{ln}\,\left(\frac{d}{r}\right)$$

For *d* = 1 μm (half a sarcomere), *I*_Na_ = 500 μA/μF (quite large), *c*_m_ = 2 μF/cm^2^, ρ = 200 Ω⋅cm, and *r* = 200 nm, one obtains 4.6 μV. With *r* = 100 nm, one obtains 6.4 μV. Therefore, for tubule radii of 100–200 nm, potential differences are in the microvolt range. While the confinement of the intracellular space by the cell membrane may somewhat increase these values, they probably remain in the sub-millivolt range, and thus negligible in comparison with transmembrane and intratubular potentials. Intuitively, this can be understood from the fact that in the intracellular space, current can flow in a relatively unhindered manner between the T-tubules, while in the tubular lumen, the current is channeled through the narrow T-tubular network. Of note, extrapolating this formula for an axon in a bath with *r* = 5 μm, *d* = 10 μm, *c*_m_ = 1 μF/cm^2^, and ρ = 200 Ω⋅cm, one obtains a potential of 35 μV, which is indeed the range registered from neuronal preparations by electrode arrays (Tscherter et al., 2016).

The model was implemented numerically using a one-dimensional finite-difference scheme. Membrane potential (*V*_m_) and the gating variables of the sodium current were integrated using the forward Euler method using a constant time step of 0.25 ns. Simulations were implemented and run in MATLAB (version 2015a, The MathWorks, Natick, MA, United States). The MATLAB source code producing the figures is provided in the Supplementary Materials.

## Results

### Delay of T-Tubular Membrane Depolarization

First, we set out to answer the question how long it takes to charge the membrane as a capacitor and depolarize the T-tubules in the absence of sodium current. In other words: what is the delay between depolarization at the plasma membrane and deep in the T-tubules?

When we consider an electrophysiological experiment in which a cardiomyocyte is voltage-clamped, a voltage step at the pipette site will first induce a capacitive current into the cell membrane, which will cause depolarization (Figure 1). While this current can travel relatively unhindered through the cytoplasm into the T-tubular membrane, the narrow T-tubules will oppose an extracellular “exit resistance” against the capacitive current as it leaves the T-tubule. The deeper and narrower the T-tubule, the higher the exit resistance, the longer it takes for the T-tubular membrane to depolarize, as shown in Figures 1B–E. The depolarization to the threshold of voltage-gated calcium channels (approx. −40 mV) proceeded along the T-tubules according to the time-vs.-position plots shown in the insets in Figures 1B–E. These time courses were characterized by monotonically increasing sigmoid curves. This delay in membrane depolarization may directly affect the ion channels in the T-tubule: the deeper in the T-tubule, the later they can open. According to our model, for a typical mouse T-tubule, the threshold of voltage-gated calcium channels in the innermost node of the T-tubule will however already be reached after ∼10 μs (Figure 1B). In a human T-tubule (Cannell et al., 2006; Jayasinghe et al., 2015), the membrane depolarization would reach the threshold of the innermost calcium channels much faster than in a murine T-tubule, after ∼2 μs (Figure 1C). In cardiac disease, T-tubules generally widen. Myocardial infarction (MI) induces an increase in T-tubular diameter of 9% in mice (Wagner et al., 2012), and as much as 33% in humans (Crossman et al., 2017b). The “exit resistance” of T-tubules therefore decreases and calcium channels deep in the T-tubule open quicker. For a murine T-tubule after MI, the delay to reach the threshold of L-type calcium channels is ∼5 μs (Figure 1D), and in a T-tubule from human post-MI, ∼0.7 μs (Figure 1E). For human T-tubules of different lengths (2 and 10 μm), membrane depolarization is shown in Supplementary Figure S1.

**FIGURE 2:**
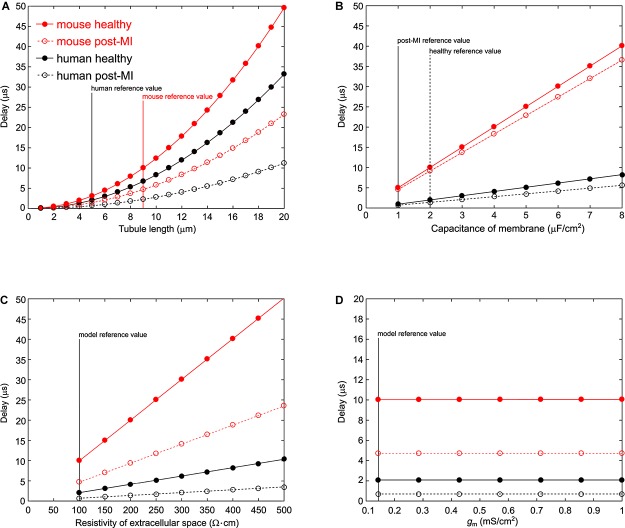
Varying parameters in the murine and human T-tubule models. In the models of mouse healthy (red solid line), mouse post-myocardial infarction (MI) (red dashed line), human healthy (black solid line), and human post-MI (black dashed line) T-tubules, we varied tubule length **(A)**, membrane capacitance **(B)**, resistivity of the extracellular space **(C)**, and membrane conductance **(D)**. The time delay after which the deepest membrane segment reached –40 mV is plotted against the respective varied parameter. Vertical lines represent reference values from the respective models.

When the tubule length was systematically varied in the murine and human T-tubular models, the delay until −40 mV was reached in the deepest T-tubular segment exhibited a non-linear dependence on tubule length (Figure 2A). When membrane capacitance or extracellular resistivity were systematically varied, the delay exhibited a linear dependency on these two parameters in all four models (Figures 2B,C). Changing membrane conductance did not affect the delay (Figure 2D). Of note, these delays always remained in the submillisecond range. The spatiotemporal profile always exhibited a sigmoid shape as shown in the insets of Figure 1.

Classical cable theory (Jack et al., 1975) can be used to estimate the voltage drop from the mouth to the end at steady state once the capacitive loading is complete. This theory assumes a homogeneous tubule immersed in an extensive conductive medium with negligible with negligible potentials outside the T-tubule, with uniform cylindrical geometry (constant diameter and cross section), specific membrane capacitance, and conductance. First, one calculates the length constant λ as

(4) $$\text{λ}=\sqrt{\frac{r}{2\,g_{\text{m}}\,{\text{ρ}}_{\text{e}}}}$$

where *r* is the T-tubular radius, *g*_m_ is the conductance of the membrane per unit area, and ρ_e_ is the resistivity of extracellular space. It follows that the length constant for the healthy mouse T-tubule (characteristics specified in Table 1) is 186 μm. One may then be tempted to use an exponential function (valid for an infinite cable) to describe the decay of membrane potential as

(5) $$\text{Δ}\,V_{\inf}\,\left(x\right)=\text{Δ}\,V_{0}\,{\text{e}}^{-\frac{x}{\text{λ}}},$$

where Δ*V*_0_ = 100 mV is the voltage applied at the tubule mouth and *x* is the distance from the tubule mouth. This approach is however erroneous, because the T-tubule has a sealed end and thus a finite length. For sealed-end cables (no current flow over the cable end), by applying the reflection and superposition principle, the correct approach is to use a hyperbolic cosine function instead of an exponential (Weidmann, 1952; Jack et al., 1975):

(6) $$\text{Δ}\,V_{\text{sealed}}\,\left(x\right)=\text{Δ}\,V_{0}\,\frac{\cosh\left(\frac{L-x}{\text{λ}}\right)}{\cosh\left(L/\text{λ}\right)}$$

For the healthy mouse T-tubule, Eq. 5 yields a voltage drop of 4.72 mV, while Eq. 6 yields only 0.117 mV. This corresponds to the negligible voltage drop shown in Figure 1B. Although the other conditions we modeled lead to different length constants (λ_mouse post–MI_ = 194 μm; λ_human healthy_ = 227 μm; λ_human post–MI_ = 276 μm), the voltage drops are negligible in all cases (mouse post-MI: Δ*V*_*inf*_ = 4.52 mV, Δ*V*_sealed_ = 0.107 mV; human healthy: Δ*V*_*inf*_ = 2.18 mV, Δ*V*_*sealed*_ = 0.0243 mV; human post-MI Δ*V*_*inf*_ = 1.7938 mV, Δ*V*_*sealed*_ = 0.0164 mV).

### Effects of T-Tubular Constrictions on Membrane Depolarization

**FIGURE 3:**
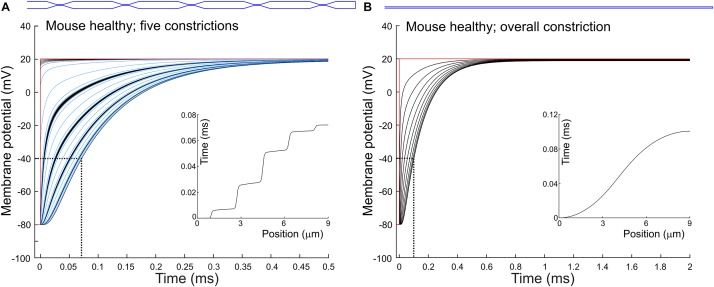
Delay of membrane depolarization in murine T-tubules with constrictions. In the healthy murine T-tubule (Figure 1B), we introduced five 10× constrictions of luminal diameter **(A)** or an overall 10× constriction **(B)**. Tubule geometries are schematically given in blue and detailed in Table 1. Membrane potentials of each **(A)** and every tenth **(B)** node are shown. Insets represent the time of depolarization to –40 mV versus position along the T-tubule.

To quantify how constrictions change the delay of depolarization deep in a T-tubule, we incorporated constrictions into our model of an unbranched cylindrical T-tubule. We used the parameters for the healthy mouse T-tubule as these parameters led to the longest depolarization delay (10 μs to depolarize the innermost T-tubule segment to −40 mV). We introduced five 450-nm-long constrictions with a 10-fold diameter reduction to 19.7 nm, their centers spaced 1.8 μm apart (Figure 3A and Table 1). This is similar to the model of Uchida and Lopatin (2018), which included 20-nm-wide constrictions every 2 μm. For this purpose, the resistances and capacitors depicted in Figure 1A were scaled accordingly. With the constrictions in our model, the threshold for Ca_v_ channels (−40 mV) in the deepest tubular segment was reached ∼75 μs later than at the surface (Figure 3A). This is 7.5 times later than in the simpler model without constrictions, where the threshold was reached after 10 μs (Figure 1B). The time after which −40 mV was reached proceeded in a staircase-like manner (Figure 3A, inset). The regions where the delay increased abruptly correspond to the constricted sites, while this threshold was reached almost synchronously in the broader regions between constrictions.

Constricting the tubule 10 times to a diameter of 19.7 nm over its full length (Figure 3B) increased the depolarization delay in the deep segment 10 times (compare Figures 1B, 3B), in agreement with cable theory (capacitive load 10 times smaller and resistance 100 times larger). Interestingly, this increase of the delay was comparable to that in the presence of five successive localized constrictions. This can be explained by the fact that in the presence of localized constrictions, each widening following each constriction represents a large capacitive load, and the accumulation of these successive loads contributes to slow the spread of electrotonic depolarization.

Additionally, we observed a voltage drop of 0.84 mV in the tubule with five constrictions (Figure 3A) and 1.2 mV in the overall constricted tubule (Figure 3B), which is ∼8 to ∼10 times higher than the voltage drop of 0.117 mV calculated for the healthy murine T-tubule.

### Implications of T-Tubular Sodium Current

**FIGURE 4:**
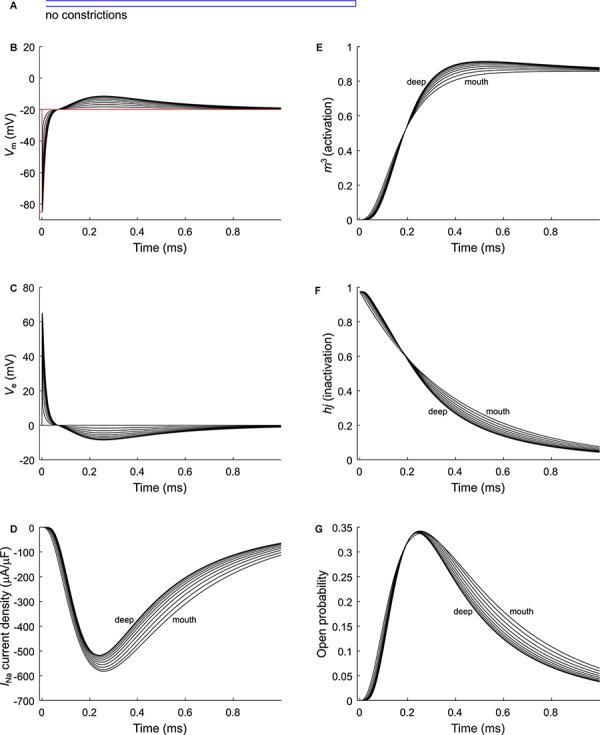
Modeling sodium current in a healthy murine T-tubule. A voltage-gated sodium current [formulated according to Luo and Rudy (1991) and Livshitz and Rudy (2009)] with a conductance of 23 mS/μF (Luo and Rudy, 1991) was introduced into the healthy mouse T-tubule model in parallel to membrane resistance (Figure 1A). **(A)** Schematic representation of the morphology of a healthy murine T-tubule (Table 1). Membrane potentials **(B)**, extracellular potentials **(C)**, and simulated sodium current density upon a voltage-clamp step of the tubule mouth from –85 to –20 mV **(D)** are given. Note the decrease of peak sodium current and delayed activation in deeper segments of the tubules **(D)**. This correlates with changes in the biophysical properties of the sodium current: product of activation gates (*m*^3^, **E**) and inactivation gates (*hj*, **F**) shows faster activation and inactivation in deeper T-tubular segments, respectively; and peak open probability (defined as *m*^3^*hj*) very slightly increases in deeper T-tubular segments **(G)**. Data are shown for every tenth node.

**FIGURE 5:**
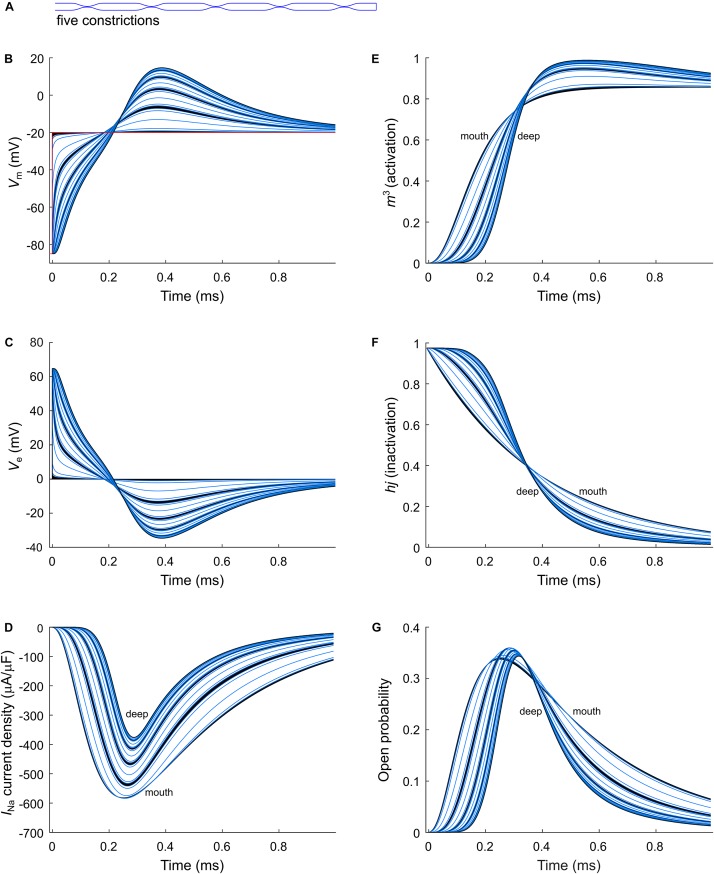
Modeling sodium current in a murine T-tubule with five constrictions. **(A)** Schematic representation of the morphology of the tubule (Table 1). Membrane potentials **(B)**, extracellular potentials **(C)**, and simulated sodium current densities upon a voltage-clamp step of the tubule mouth from –85 to –20 mV **(D)** are given. The peak sodium current decreases in deeper tubular segments, while activation is delayed and inactivation is faster **(D)**. This correlates with a lower driving force **(B)**, and changes in the activation (*m*^3^, **E**) and inactivation gates (*hj*, **F**). Peak open probability (defined as *m*^3^*hj*) slightly increases in proximal T-tubular segments and then decreases in deeper T-tubular segments **(G)**. Curve colors represent luminal diameter from largest (black) to smallest (light blue). Data are shown for each node.

**FIGURE 6:**
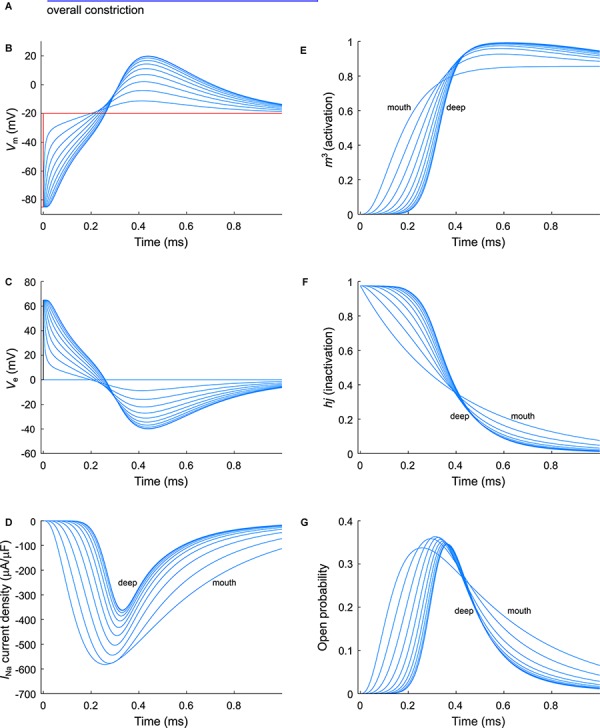
Modeling sodium current in an overall constricted murine T-tubule. **(A)** Schematic representation of the morphology of the tubule (Table 1). Membrane potentials **(B)**, extracellular potentials **(C)**, and simulated sodium current densities upon a voltage-clamp step of the tubule mouth from –85 to –20 mV **(D)** are given. The sodium current decreases in deeper segments, while activation is delayed and inactivation is faster in deeper segments of the tubules **(D)**. This correlates with a lower driving force **(B)**, and changes in the activation gates (*m*^3^, **E**) and inactivation gates (*hj*, **F**). Peak open probability (defined as *m*^3^*hj*) slightly increases in proximal T-tubular segments and then decreases in deeper T-tubular segments, **G**). Data are shown for every tenth node.

As a next step, we investigated the effect of putative voltage-gated sodium (Na_v_) channels on tubular depolarization (see Table 1 for parameters). Results using the Luo–Rudy–Livshitz *I*_Na_ model in the non-constricted T-tubule are presented in Figure 4. Figure 4D shows that in a non-constricted T-tubule, the sodium current is smaller deep inside the T-tubule than at the mouth. This smaller current is due to the appearance of a negative extracellular potential in the T-tubule (Figure 4C), which results from the flow of current along the narrow tubule. This current is necessary to compensate the charge being lost in the tubule lumen due to *I*_Na_ (principle of charge conservation). The negative extracellular potential contributes to the depolarization of the membrane a few mV beyond −20 mV (Figure 4B). Since constrictions have been shown to be an important determinant governing electrical properties (Uchida and Lopatin, 2018), we also introduced a sodium current in the T-tubule with five constrictions and the T-tubule constricted over its entire length (Figures 5, 6). The extracellular potential reaches up to −35 and −40 mV in the tubule with five constrictions and the overall constricted tubule, respectively (Figures 5C, 6C). Importantly, this negative extracellular potential brings the transmembrane potential closer to the Nernst potential of sodium (*E*_Na_ = 55 mV), which diminishes the driving force of the sodium current (*V*_m_–*E*_Na_) and thus the sodium current itself (∼15% reduction in an unconstructed T-tubule, ∼35% in a T-tubule with five constrictions, and ∼40% in an overall constricted T-tubule) (Figures 4D, 5D, 6D). Thus, the sodium current is smaller in deeper T-tubular membrane segments.

Constricting the T-tubule delays the onset of the sodium current from ∼0.03 (no constrictions) to ∼0.15 ms (overall constriction) and ∼2 ms (five constrictions) (Figures 4D, 5D, 6D). Moreover, we observed changes in sodium current biophysical properties in deep segments compared to surface segments of all three tubular models: faster activation (Figures 4E, 5E, 6E), inactivation (Figures 4F, 5F, 6F), and an increase in open probability, especially in proximal segments (Figures 4G, 5G, 6G). These effects were more pronounced in the overall constricted tubule and tubule with five constrictions than in the unconstricted tubule. Expectedly, the magnitude of the aforementioned effects depends on T-tubular length. Supplementary Figures S2–S4 show that in 5.4-μm-long tubules without constrictions, with three constrictions, or with an overall constriction, a T-tubular sodium current affects extracellular potential, sodium current attenuation, and sodium current biophysical properties in a similar manner but to a lesser extent than in the 9-μm-long tubules (Figures 4–6).

**FIGURE 7:**
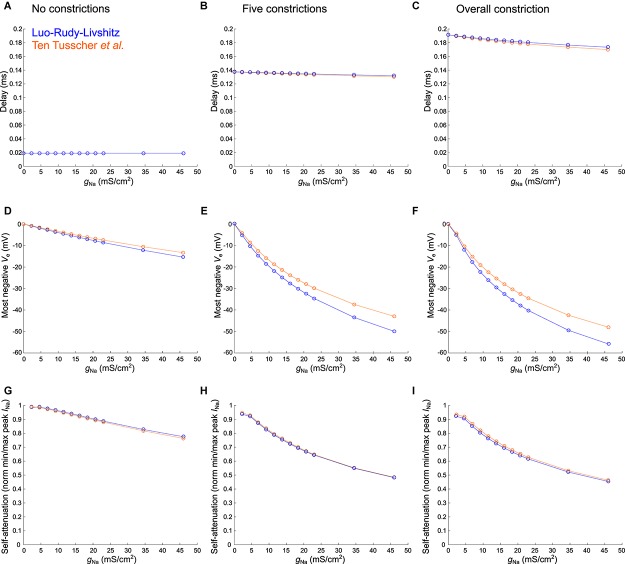
Varying sodium current parameters in the healthy murine T-tubular model with and without constrictions. The Luo–Rudy–Livshitz sodium current (*I*_Na_) model [blue (Luo and Rudy, 1991; Livshitz and Rudy, 2009)] is compared to the ten Tusscher et al. (2004) *I*_Na_ model (orange). For the tubule without constrictions **(left)**, the tubule with five constrictions **(middle)**, and the overall constricted tubule **(right)**, the plots show the delay after which –40 mV is reached in the innermost segment (**A–C**; in panel **A**, both curves overlap), the most negative extracellular potential *V*_e_ **(D–F)**, and the quantification of self-attenuation **(G–I)** versus the maximal sodium current conductance *g*_Na_. *g*_Na_ was systematically varied using the following values: 2.3, 4.6, 6.9, 9.2, 11.5, 13.8, 16.1, 18.4, 20.7, 23, 34.5, and 46 mS/μF. Self-attenuation was quantified as the smallest peak *I*_Na_ in the tubule (in absolute value) normalized by the largest peak *I*_Na_.

Results using the *I*_Na_ model by ten Tusscher et al. (2004) instead of the Luo–Rudy–Livshitz model in the same tubular models are shown in Supplementary Figures S5–S7. The results using the Luo–Rudy–Livshitz (Figures 4–6) and ten Tusscher et al.’s (2004) (Supplementary Figures S5–S7) models were very similar. The main differences lie in the lower peak open probability with the ten Tusscher et al.’s (2004) model compared to the Luo–Rudy–Livshitz model. This smaller open probability is related to a different behavior of steady state inactivation: at −85 mV, more sodium channels are inactivated in the ten Tusscher et al.’s (2004) model than in the Luo–Rudy–Livshitz model (compare for instance Figure 4F to Supplementary Figure S5F). This led to a decreased *I*_Na_ density and a less negative extracellular potential. However, the degree of sodium current self-attenuation was similar (Figures 7G–I).

In Figure 7, we explored the effects of systematically varying the maximal sodium current conductance (*g*_Na_) and the sodium current model on the delay after which −40 mV is reached, the minimal extracellular potential and on *I*_Na_ self-attenuation. Firstly, *g*_Na_ hardly affects the delay to −40 mV in the deepest segment of the T-tubules without and with constrictions (Figures 7A–C). However, a larger *g*_Na_ induces a more negative extracellular potential (Figures 7D–F) and a greater level of self-attenuation (Figures 7G–I). This is logical as an increase in sodium conductance leads to a greater sodium current density, while the concomitant increased axial current along the T-tubule enhances the negative extracellular potential and self-attenuation.

The choice of sodium current model hardly influences the time after which −40 mV is reached (Figures 7A–C). Changing *g*_Na_ however affects extracellular potentials stronger in the Luo–Rudy–Livshitz model than in the ten Tusscher et al.’s (2004) model (Figures 7D–I), as the peak open probability is smaller in the ten Tusscher et al.’s (2004) model, which decreases sodium current density and extracellular potential.

## Discussion

This work addressed hitherto incompletely explored passive and active electrical properties of T-tubules. We show that (1) the depolarization delay in deep T-tubular membrane is negligible in our models; (2) the voltage drop along a passive T-tubule is negligible; (3) the depolarization delay depends mainly on tubule length; and (4) sodium current interacts with intraluminal potentials, which modifies its kinetics and makes it smaller (self-attenuation) in deep T-tubular segments in a *g*_Na_-dependent fashion.

The results of our computational model show that the delay in T-tubular depolarization and T-tubular sodium current depend on the exact geometry of the T-tubule, notably on spatial variations in tubule diameter and the presence of constrictions. The exact location of the constrictions are also expected to modulate these factors.

We observed that the activation threshold of L-type voltage-gated calcium channels in deep T-tubular segments is attained within a very short time after the cell surface is excited when the “exit resistance” of capacitive current is taken into account (∼0.01 ms for a mouse T-tubule without constrictions and up to ∼0.10 ms with overall constriction). Thus, the delay of depolarization of mouse and human T-tubules is sufficiently short to ensure excitation–contraction coupling. The T-tubular delay represents no major latency for the sodium current considering that the conduction time along a 100-μm-long cell with a macroscopic conduction velocity of 100 cm/s would be 100 μs (Rohr, 2004). It must be noted that T-tubular length probably greatly varies *in vivo*, with concomitant changes in depolarization time. The deepest segment of a 20-μm-long healthy mouse T-tubule without constrictions however would depolarize after 50 μs, which is still negligible (Figure 2A).

Human T-tubules depolarize quicker than murine T-tubules because they are relatively wide and short. On a cross-section of a cardiomyocyte, human T-tubules look like spokes on a wheel, and do not form intricate networks like in murine cells (Jayasinghe et al., 2015). The depolarization delay in human T-tubules is therefore negligible in this model. When adapting our model to simulate remodeled T-tubules after MI, the activation threshold of calcium channels is reached even faster due to the loss of microfolds (Hong et al., 2014) and increase of luminal diameter (Wagner et al., 2012; Crossman et al., 2017b). The T-tubular widening might reduce the relative depletion of calcium and accumulation of potassium in the restricted T-tubular lumen, affecting the driving force of their respective ion channels (Hong and Shaw, 2017). The disturbed calcium cycling associated with disease should have other causes, such as dyad uncoupling (Song et al., 2006) in conjunction with reduced expression of junctophilin-2, which is involved in dyad stability (Reynolds et al., 2016), and of Bin1, which shapes the T-tubular membrane and aids Ca_v_1.2 trafficking (Hong et al., 2010; Fu and Hong, 2016). Physical obstruction of T-tubules has also been observed in a murine heart failure model (Scardigli et al., 2017).

Without dilatations and constrictions, our model gives a negligible voltage drop of 0.117 mV (or 0.117%) at steady state from mouth to deep T-tubular node. This is comparable to previously reported results: for a rat T-tubule of 6.84 μm (Soeller and Cannell, 1999), Scardigli et al. (2017) calculated a length constant of 290 ± 90 μm and a voltage drop from the surface sarcolemma to the core of ∼4 mV. Scardigli et al. (2017) however applied the equation for an infinite cable (Eq. 5); applying Eq. 6 for a sealed cable gives a voltage drop of 0.028 mV. A slightly larger but still minuscule value is found for the considerably smaller length constant of 68 μm derived by Uchida and Lopatin (2018) in a finite-element model of dextran diffusion in branched T-tubules with constrictions. When assuming a murine T-tubule of 9 μm, the voltage drop would be 13 mV for an infinite cable and 0.870 mV for a sealed-end cable and is therefore still negligible. However, care must be taken when interpreting these values, since classic linear cable theory cannot be applied straightforwardly to morphologically heterogeneous T-tubules. Indeed, the assumptions of a uniform cylindrical geometry and uniform membrane properties have a limited validity in the context of T-tubules, which exhibit variations of diameter.

Moreover, tubules exhibit branching, which we did not consider. Several confocal and super-resolution imaging studies have demonstrated the complex T-tubular topology (Soeller and Cannell, 1999; Hong et al., 2014; Jayasinghe et al., 2015; Fu and Hong, 2016; Scardigli et al., 2017). T-tubular branching in rodents occurs roughly every sarcomere length (Savio-Galimberti et al., 2008). Therefore, the T-tubules we modeled may not reflect the complexity of the T-tubular network, and likely do not occur as such in any cardiomyocyte. Future efforts should assess the effects of the complex geometry and topology of branches in tubular networks, and systematically evaluate branch patterns, branch lengths, branch intervals, heterogeneities in branch lengths and intervals, more elaborate branch geometries, and the occurrence of loops. Uchida and Lopatin (2018) addressed some of the effects of constrictions and branches, and concluded that constrictions mainly govern electrical behavior, while branches only have relatively small effects. Therefore, we focused on constrictions in our study. To evaluate the behavior of membrane potential in T-tubules with constrictions, we recoursed to our model as, to our knowledge, no general formula exists to calculate the decay of potential along an irregular structure. We observed in our simulation of a T-tubule with five constrictions a voltage drop of 0.84 mV. Although this voltage drop is about eight times higher than in the non-constricted tubule, it does not affect the opening of voltage-gated channels, which open at much lower potentials.

Our model is considerably simpler than a number of previous models of T-tubules. The model of Kekenes-Huskey et al. (2012) incorporated one tubule with the L-type calcium current, the sodium–calcium exchange current, the calcium pump current and the calcium leak current. The model also incorporated calcium diffusion. However, the model did not incorporate dynamic changes of membrane potential. The model of Hatano et al. (2015) is an extremely sophisticated model incorporating, in addition to intracellular, extracellular, and membrane potentials, contraction dynamics, and fluid movement in the tubules. This model would certainly be appropriate to address the questions posed in the present study. However, implementing this model requires highest expertise, and to our knowledge, no source code is available. The EMI (Extracellular space, Membrane, Intracellular space) model by Jæger et al. (2019) also incorporates extracellular, intracellular, and membrane potentials explicitly, but it handles only rectangular geometries due to the finite-difference scheme used in the computations. The model by Oliveira and Rodrigues (2019) is a detailed model coupling calcium concentration, contraction, and cell deformation, but electrical potentials are not represented.

In addition, in our model, we did not incorporate irregular tubular shapes (e.g., non-circular or variable cross-section shapes, highly convolved tubules) or ion concentration changes. The first would require a finite-element modeling approach and the second would require the implementation of ion fluxes using the Nernst–Planck equation. Both are numerically much more elaborate and demanding. Rather, our aim was to develop a model answering the following two questions: (1) how long does it take for a depolarization to propagate into the T-tubule and (2) how does the presence of the sodium current influence this depolarization. The simplicity of our model can be regarded as an advantage as it permits new insights with modest computational effort.

When inserting the sodium current in our computational model, we found that the sodium current self-attenuates deep in the T-tubules, and the extracellular potential becomes negative (Figures 4–6). This is explained by the very small T-tubule diameter. At the cardiac intercalated disc, where two opposing membranes are also very close together, a similar effect has been predicted (Rhett et al., 2013; Veeraraghavan et al., 2015; Hichri et al., 2018). Importantly, sodium depletion in the T-tubule caused by sodium entering the cell may even augment the sodium current self-attenuation by decreasing the Nernst potential of sodium. Such a phenomenon has been modeled computationally at intercalated discs, but the influence of extracellular potentials nevertheless prevails over sodium depletion (Mori et al., 2008).

The self-attenuation of the sodium current will not affect the calcium current as this effect dies out before the calcium channels open. Moreover, we assumed stable calcium concentrations during our simulations, as we focused on the characterization of the sodium current in our study and ran short simulations of 1 ms, given that the sodium current lasts about 1 ms (Figures 4–6). During this time window, calcium channels will not have activated significantly yet. Furthermore, during the first millisecond, the sodium–calcium exchange current will hardly contribute to depolarization. The sodium–calcium exchange current may be relevant at a later time point for the ion dynamics in dyads, but our model did not incorporate the latter explicitly.

Interestingly, sodium current showed faster activation and inactivation kinetics in the deeper segments of the tubule due to the negative extracellular potentials, and the resulting more positive transmembrane potentials. Given that length and constrictions influence the sodium current, changing the T-tubular geometrical pattern, for instance during heart failure (Wagner et al., 2012; Crossman et al., 2017b; Scardigli et al., 2017), may affect macroscopic conduction. This remains widely unexplored.

Given the self-attenuation of the sodium current, it may be interesting to investigate whether the late sodium current in deep T-tubular segments is also quenched, which has been predicted to occur at the intercalated disc (Greer-Short et al., 2017). This process might not play a substantial role in human cardiac arrhythmias, as human T-tubules are wider and shorter than the murine tubules we modeled here. However, first and foremost, the T-tubular sodium current density needs to be reliably determined in murine and human cells to approach extracellular potentials and sodium current self-attenuation with *in silico* models as realistically as possible. We might place hope in the further development of the scanning ion conductance technique, which has yet only been able record currents at the mouth of the T-tubule (Bhargava et al., 2013; Rivaud et al., 2017). We might also place hope in the development of novel optogenetic voltage reporters that would be specifically expressed in T-tubules.

Importantly, the Luo–Rudy–Livshitz model [developed initially for the guinea pig action potential (Luo and Rudy, 1991; Livshitz and Rudy, 2009)] and the ten Tusscher–Noble–Noble–Panfilov model [developed for the human action potential (ten Tusscher et al., 2004)] led to similar results. We thus do not expect any major qualitative difference with other models of the sodium current (or models for other species).

It would of course be interesting to see how the results are influenced when the intracellular potential is part of the dynamics. Such an investigation would require a detailed model of the extracellular space, the intracellular space and the membrane, such as the finite element model of Hatano et al. (2015), the aforementioned EMI model (Jæger et al., 2019), or using the framework of Agudelo-Toro and Neef (2013). However, based on the considerations given in the section “Materials and Methods,” we expect intracellular potentials and gradients and thus their influences to be small. We believe that more advanced modeling would yield qualitatively similar results. Moreover, as long as the presence and relevance of voltage-gated sodium channels in T-tubules remain controversial (Rougier et al., 2019), our T-tubular sodium current model is only hypothetical. Should the presence of functional sodium channels be confirmed in the future, it will become of interest to develop such models to obtain a more comprehensive picture.

Taken together, our study gives important insights in the passive and active electrical behaviors of cardiac T-tubules. We show that biophysical properties of the sodium current as well as T-tubular depolarization greatly depend on T-tubular geometry. When investigating T-tubular structure and function in health and disease, considering these behaviors may be worthwhile to understand the functional consequences of structural remodeling, especially in context of the T-tubular sodium current.

## Data Availability Statement

All datasets generated for this study and the respective source codes are included in article/Supplementary Material.

## Author Contributions

SV contributed to the conceptualization, the preparation and the writing of the original draft. SV and JK contributed to the methodology and visualization. SV, HA, and JK contributed to the reviewing and editing of the manuscript. HA and JK contributed to the supervision. JK contributed to the software and formal analysis.

## Conflict of Interest

The authors declare that the research was conducted in the absence of any commercial or financial relationships that could be construed as a potential conflict of interest.
