# Supplementary material for: Modeling Depolarization Delay, Sodium Currents, and Electrical Potentials in Cardiac Transverse Tubules
Source: Front Physiol. 2019 Dec 10;10:1487. doi: 10.3389/fphys.2019.01487 (PMC6916517; doi:10.3389/fphys.2019.01487)
Supplement: Supplementary file 1 [file Data_Sheet_1.zip › Documentation.pdf]

Modeling depolarization delay, sodium currents, and electrical potentials in cardiac transverse tubules

Vermij S.H., Abriel H., Kucera J.P.

MATLAB source code generating the curves shown in the figures.
Validated and ran on version 2015b

Functions:

`mhjLR1_livshitz`
Function computing the gating functions of the gates m, h and j according to Luo and Rudy (1991) and Livshitz and Rudy (2009)

`mhjTNNP`
Function computing the gating functions of the gates m, h and j according to Ten Tusscher, Noble, Noble and Panfilov (2004)

`fTtubule`
Core function running the simulations.
For changing the parameters of the model, please refer to the detailed description of inputs and outputs at the beginning of the source code of this function.

Scripts:

`Fig1B_main`
Generates the plots in Fig. 1B.

`Fig1C_main`
Generates the plots in Fig. 1C. Adjust the x and y ranges if necessary.

`Fig1D_main`
Generates the plots in Fig. 1D.

`Fig1E_main`
Generates the plots in Fig. 1E. Adjust the x and y ranges if necessary.

`Fig2A_varyL_main`
Generates one curve in Fig. 2A. To obtain the different curves, adjust the variables “species” and “status” as appropriate.

`Fig2B_varyCm_main`
Generates one curve in Fig. 2B. To obtain the different curves, adjust the variables “species” and “status” as appropriate. Adjust the x and y ranges if necessary.

`Fig2C_varyRho_main`
Generates one curve in Fig. 2C. To obtain the different curves, adjust the variables “species” and “status” as appropriate. Adjust the x and y ranges if necessary.

`Fig2D_varyGm_main`
Generates one curve in Fig. 2D. To obtain the different curves, adjust the variables “species” and “status” as appropriate. Adjust the x and y ranges if necessary.

`Fig3A_main`
Generates the plots in Fig. 3A.

`Fig3B_main`
Generates the plots in Fig. 3B.

`Fig4_main`
Generates the plots in Fig. 4. Adjust the y ranges if necessary.

`Fig5_main`
Generates the plots in Fig. 5. Adjust the y ranges if necessary.

`Fig6_main`
Generates the plots in Fig. 6. Adjust the y ranges if necessary.

`Fig7_main`
Generates one set of curves (delay, most negative V_e and self-attenuation) in Fig. 7. To obtain the different curves, adjust the following variables:

INaModel to either "mhjLR1_livshitz" or "mhjTNNP"

Pattern to "No constrictions", "Overall constriction" or "5 constrictions"

Adjust the axes ranges if necessary.

`FigS1A_main`
Generates the plots in Fig. S1A. Adjust the x and y ranges if necessary.

`FigS1B_main`
Generates the plots in Fig. S1B. Adjust the x and y ranges if necessary.

`FigS1C_main`
Generates the plots in Fig. S1C. Adjust the x and y ranges if necessary.

`FigS1D_main`
Generates the plots in Fig. S1D. Adjust the x and y ranges if necessary.

`FigS2_main`
Generates the plots in Fig. S2. Adjust the x and y ranges if necessary.

`FigS3_main`
Generates the plots in Fig. S3. Adjust the x and y ranges if necessary.

`FigS4_main`
Generates the plots in Fig. S4. Adjust the x and y ranges if necessary.

`FigS5_main`
Generates the plots in Fig. S5. Adjust the x and y ranges if necessary.

`FigS6_main`
Generates the plots in Fig. S6. Adjust the x and y ranges if necessary.

`FigS7_main`
Generates the plots in Fig. S7. Adjust the x and y ranges if necessary.
