# Supplementary material for: Modeling Depolarization Delay, Sodium Currents, and Electrical Potentials in Cardiac Transverse Tubules
Source: Front Physiol. 2019 Dec 10;10:1487. doi: 10.3389/fphys.2019.01487 (PMC6916517; doi:10.3389/fphys.2019.01487)
Supplement: Supplementary file 2 [file Image_1.pdf]

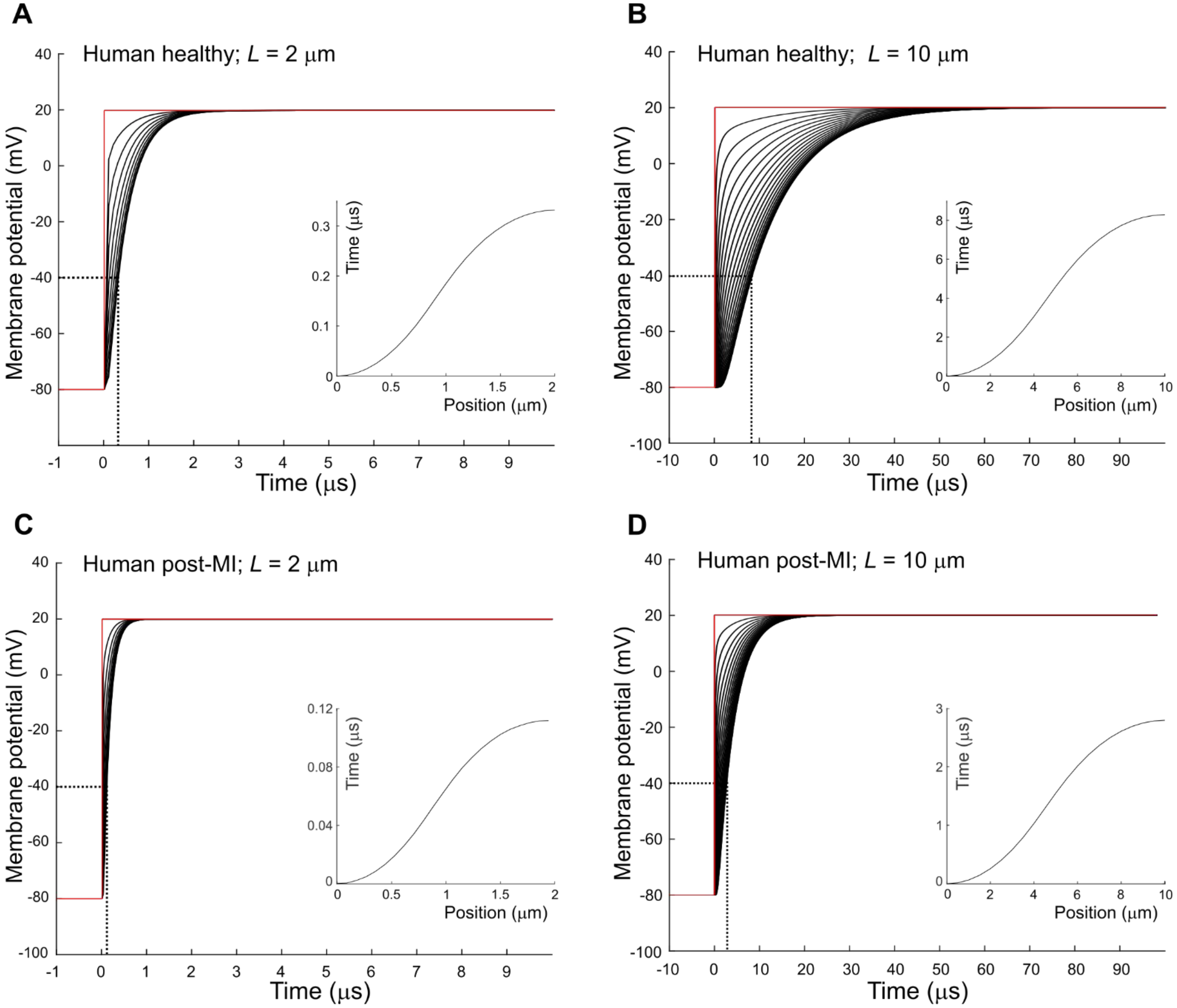

Supplemental figure 1. Membrane depolarization delay in human T-tubules of different lengths. Human T-tubules were modeled according to values given in **Table 1** but with different lengths: human healthy, *L* = 2 μm (**A**) and *L* = 10 μm (**B**); human post-myocardial infarction (MI), *L* = 2 μm (**C**) and *L* = 10 μm (**D**). A voltage step from -80 mV to +20 mV was applied to the mouth of the T-tubule (red lines in panels **A-D**; see **Figure 1A**), and membrane potentials are given for every second node (black curves in panels **A-D**). Dotted lines indicate opening threshold for voltage-gated calcium channels (around -40 mV). Insets represent the time of depolarization to -40 mV versus position along the T-tubule.

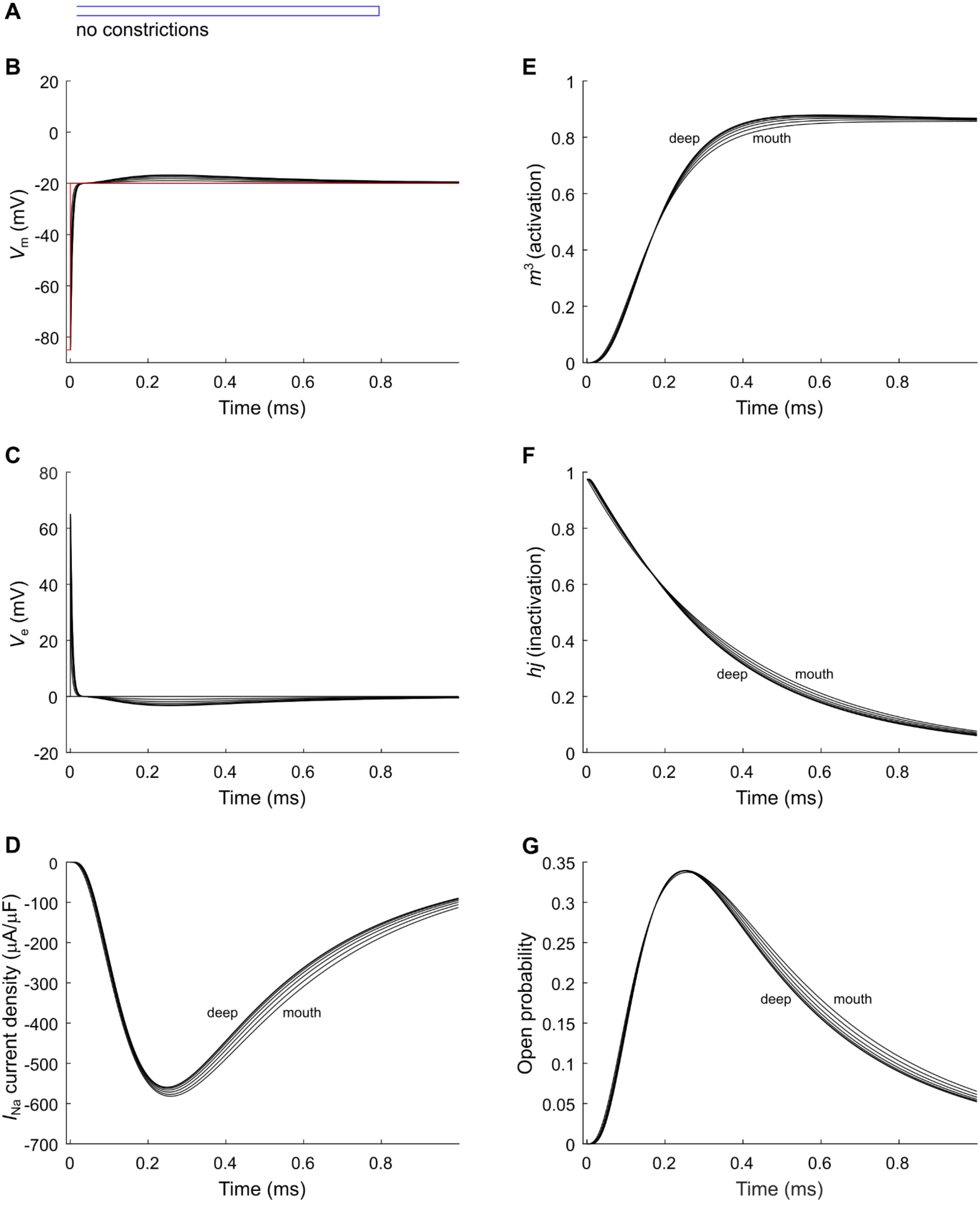

Supplemental figure 2. Modeling sodium current in a 5.4-µm-long healthy murine T-tubule. A voltage-gated sodium current (formulated according to (Luo and Rudy, 1991; Livshitz and Rudy, 2009)) with a conductance of 23 mS/µF (Luo and Rudy, 1991) was introduced in parallel to membrane resistance into a healthy mouse T-tubule model with L = 5.4 µm (compare to **Figure 4** where L = 9 µm). (**A**), Schematic representation of the morphology of the T-tubule. Membrane potentials (**B**), extracellular potentials (**C**), and simulated sodium current density upon a voltage-clamp step of the tubule mouth from -85 to -20 mV (**D**) are given. Panels **E-G** show the biophysical properties of the sodium current, including activation gates (m^3, **E**), inactivation gates (hj, **F**), and open probability (defined as m^3hj, **G**). Data are shown for every tenth node.

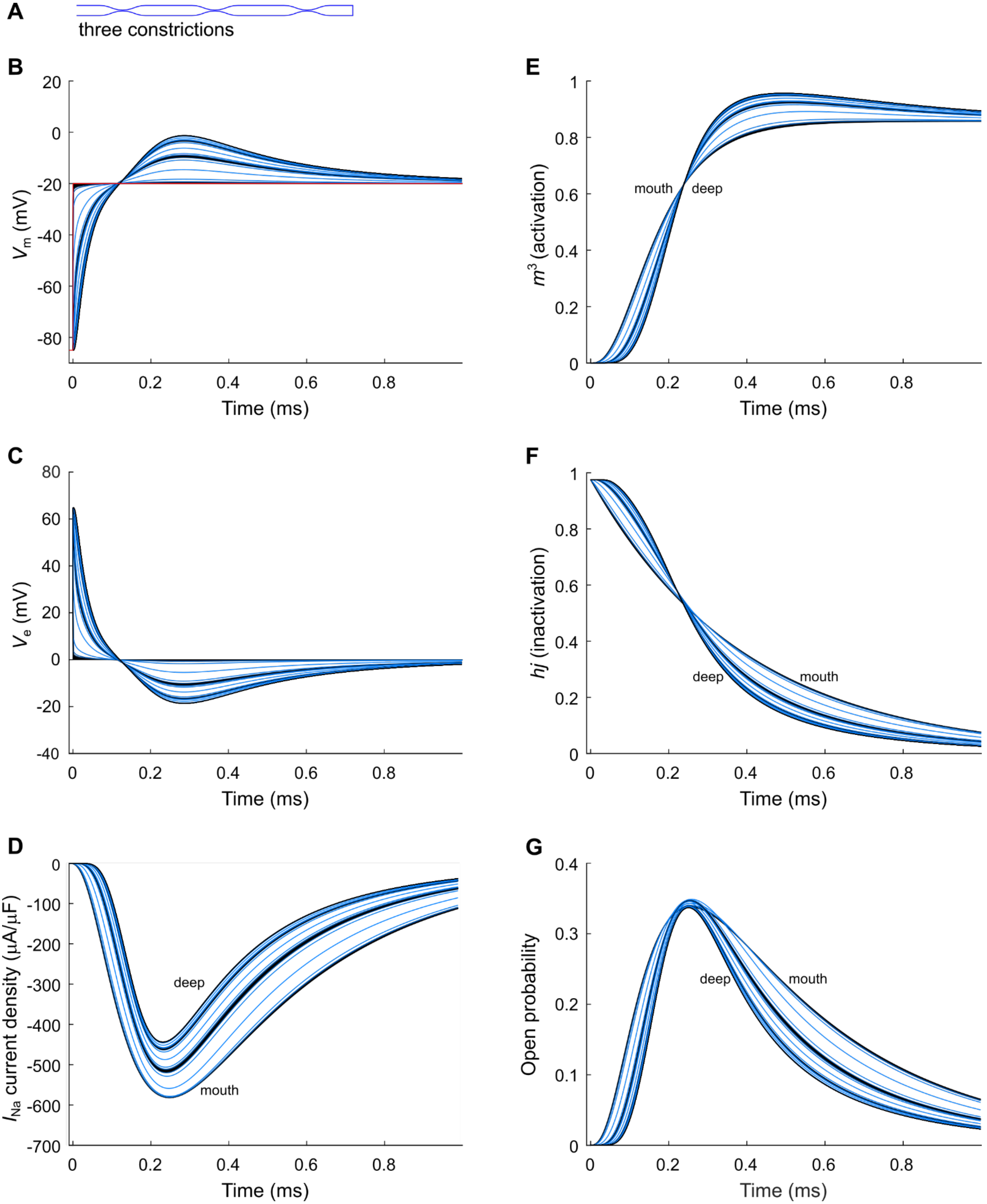

Supplemental figure 3. Modeling sodium current in a 5.4-µm-long murine T-tubule with three constrictions. (**A**), Schematic representation of the morphology of the T-tubule. Membrane potentials (**B**), extracellular potentials (**C**), and simulated sodium current density upon a voltage-clamp step of the tubule mouth from -85 to -20 mV (**D**) are given. Panels **E-G** show the biophysical properties of the sodium current, including activation gates (m^3, **E**), inactivation gates (*hj*, **F**), and open probability (defined as m^3hj, **G**). Curve colors represent luminal diameter from largest (black) to smallest (light blue). Data are shown for each node. Compare to **Figure 5**, where a 9-µm-long T-tubule with five constrictions is modeled.

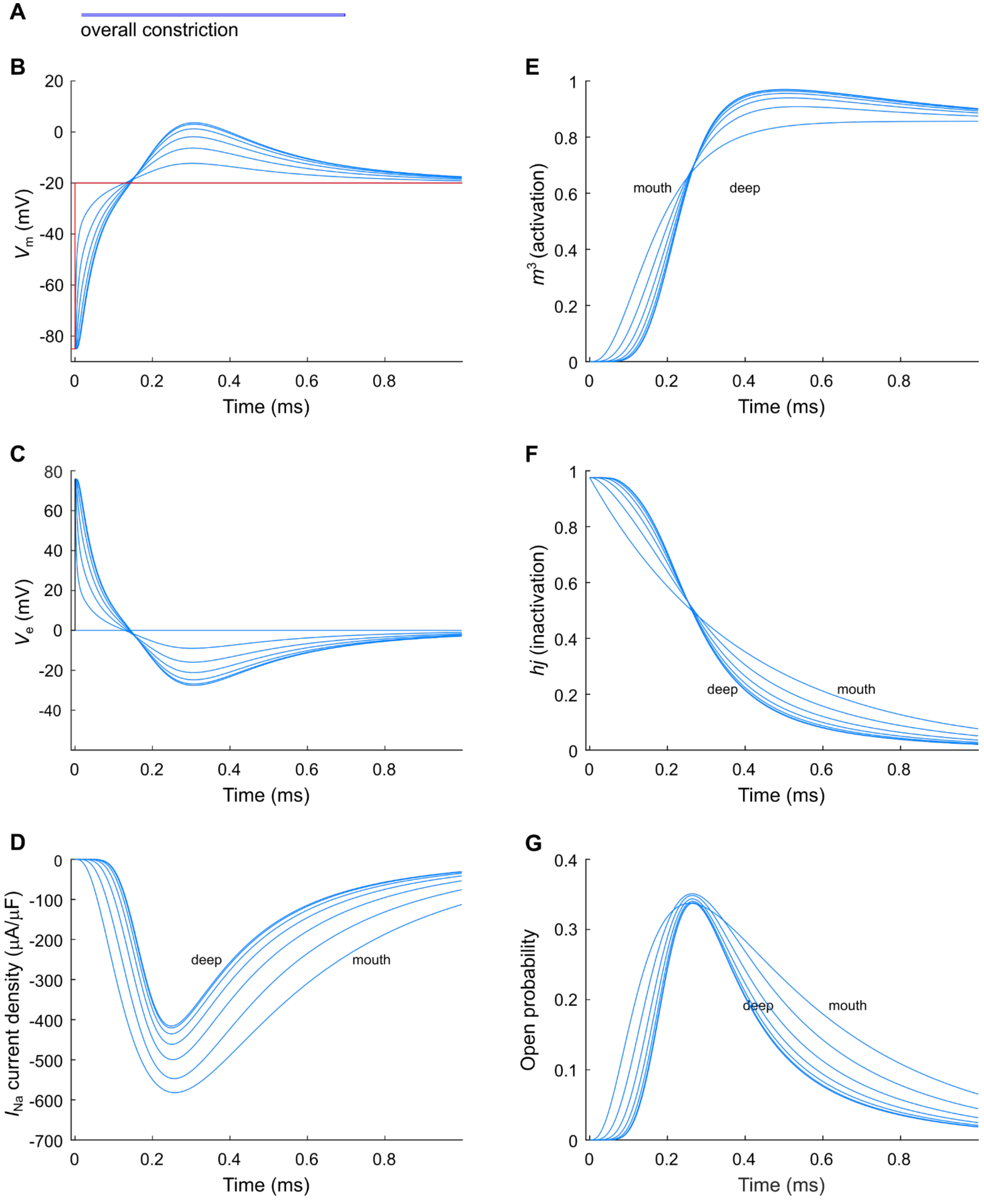

Supplemental figure 4. Modeling sodium current in an 5.4-µm-long overall constricted murine T-tubule. (**A**), Schematic representation of the morphology of the tubule (compare to **Figure 6** where L = 9 µm). Membrane potentials (**B**), extracellular potentials (**C**), and simulated sodium current density upon a voltage-clamp step of the tubule mouth from -85 to -20 mV (**D**) are given. Panels **E-G** show the biophysical properties of the sodium current, including activation gates (m^3, **E**), inactivation gates (hj, **F**), and open probability (defined as m^3hj, **G**). Data are shown for every tenth node.

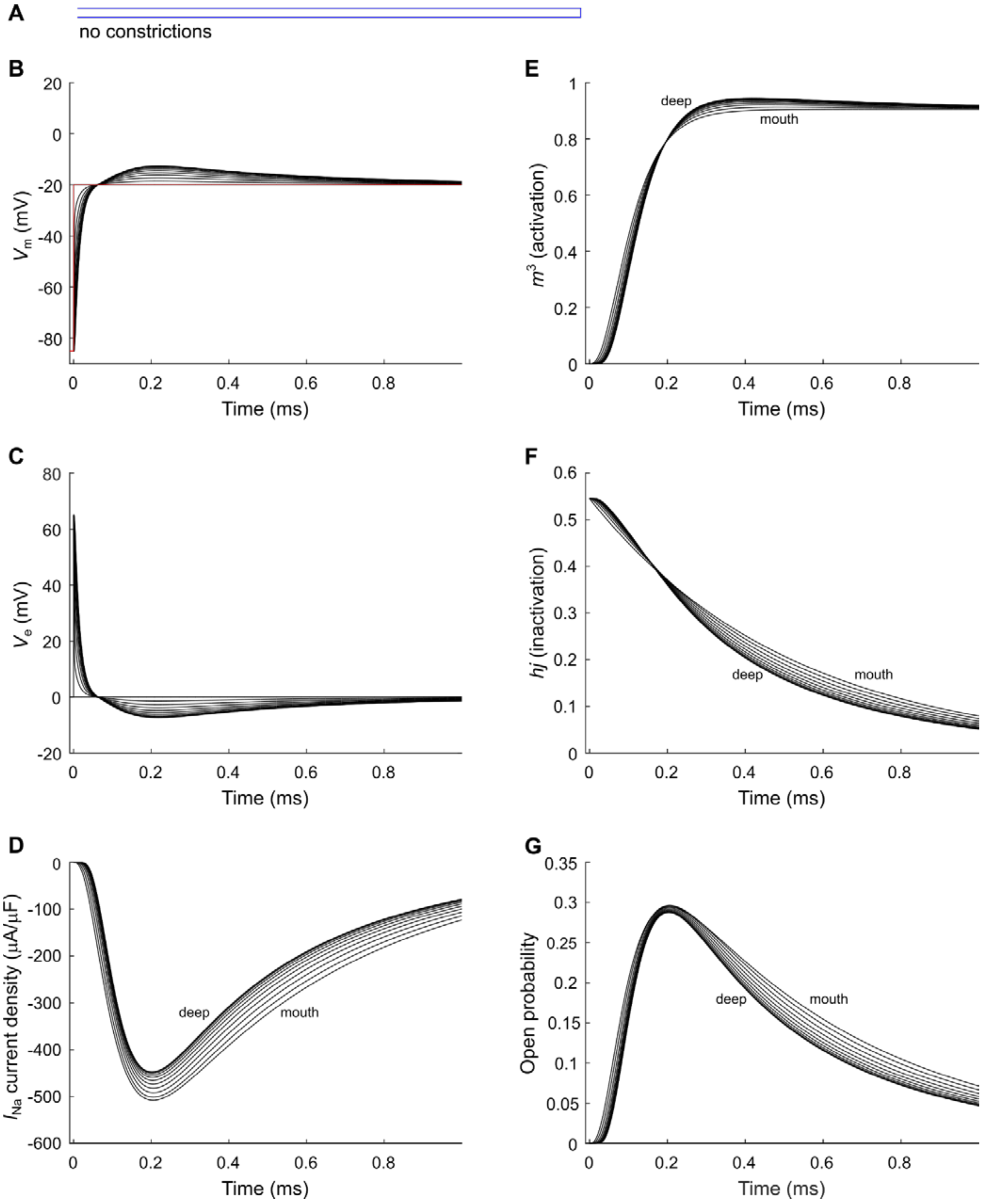

Supplemental figure 5. The Ten Tusscher *et al.* sodium current model from in a healthy murine T-tubule. The voltage-gated sodium current model by Luo-Rudy- Livshitz used for **Figure 4** (Luo and Rudy, 1991; Livshitz and Rudy, 2009) was replaced by the Ten Tusscher *et al*. model (ten Tusscher et al., 2004)). Sodium current conductance was kept at 23 mS/μF. (**A**), Schematic representation of the morphology of a healthy murine T-tubule (see **Table 1**). Membrane potentials (**B**), extracellular potentials (**C**), and simulated sodium current density upon a voltage-clamp step of the tubule mouth from -85 to -20 mV (**D**) are given. Note the decrease of peak sodium current and delayed activation in deeper segments of the tubules (**D**). This correlates with changes in the biophysical properties of the sodium current: product of activation gates (m^3, **E**) and inactivation gates (hj, **F**) show faster activation and inactivation in deeper T-tubular segments, respectively; and peak open probability (defined as m^3hj) slightly decreases in deeper T-tubular segments (**G**). Data are shown for every tenth node.

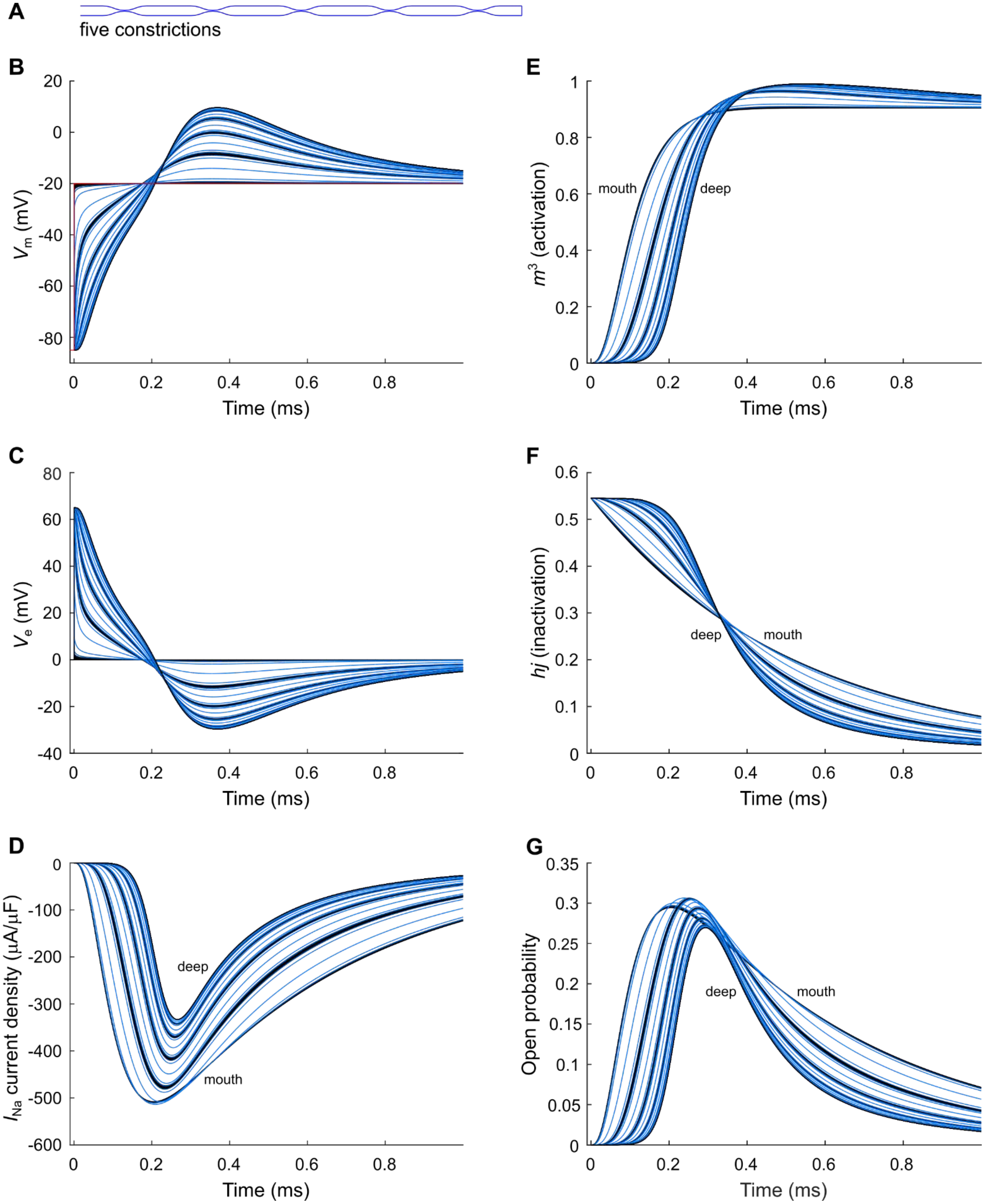

Supplemental figure 6. The Ten Tusscher *et al.* sodium current model in a healthy murine T-tubule with five constrictions. (**A**), Schematic representation of the morphology of the tubule (see **Table 1**). Membrane potentials (**B**), extracellular potentials (**C**), and simulated sodium current density upon a voltage-clamp step of the tubule mouth from -85 to -20 mV (**D**) are given. The peak sodium current decreases in deeper tubular segments, while activation is delayed and inactivation is faster (**D**). This correlates with a lower driving force (**B**), and changes in the activation (m^3, **E**) and inactivation gates (*hj*, **F**). Peak open probability (defined as m^3hj) very slightly increases in proximal T-tubular segments and then decreases in deeper T-tubular segments (**G**). Curve colors represent luminal diameters from largest (black) to smallest (light blue).

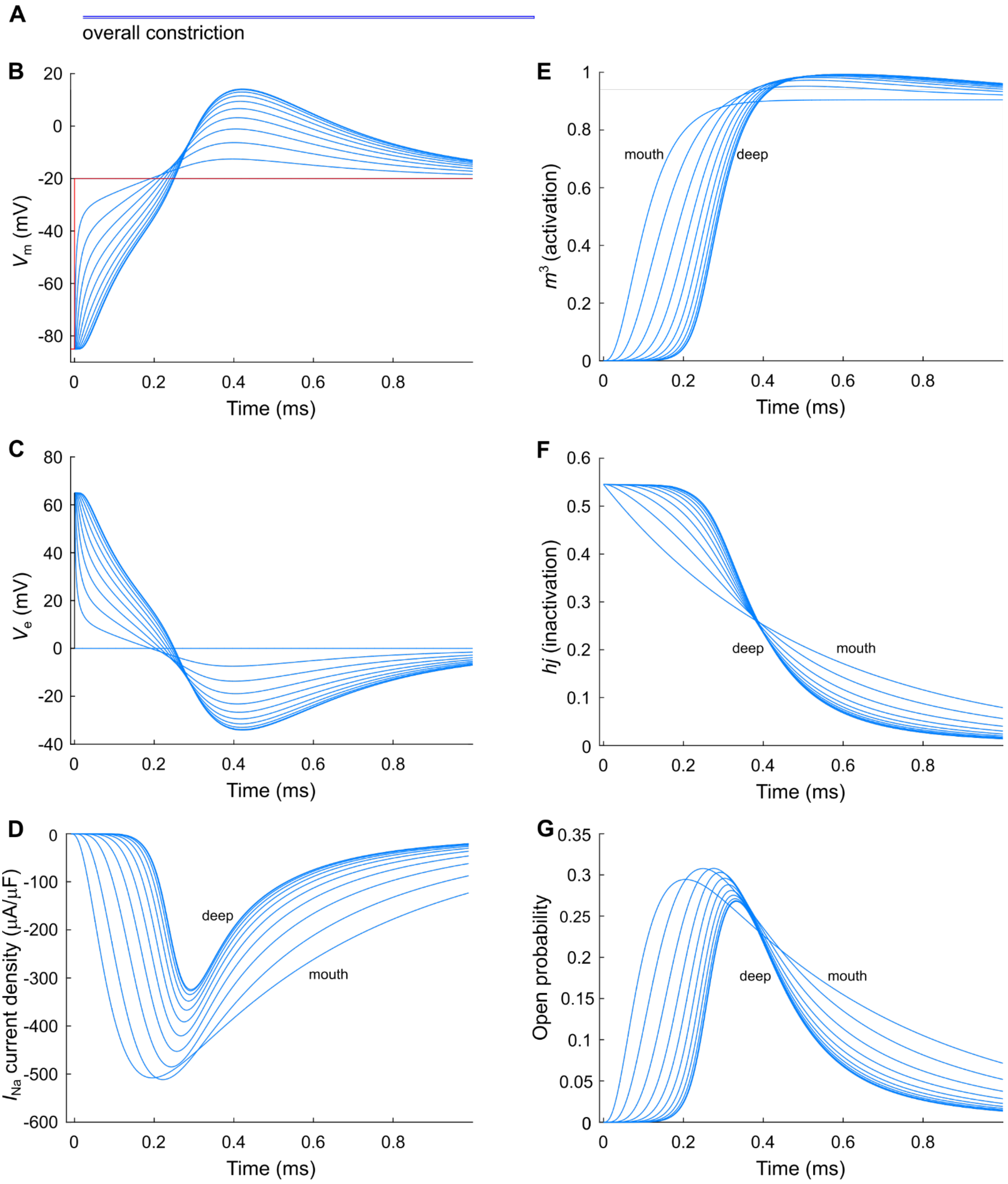

Supplemental figure 7. The Ten Tusscher *et al.* sodium current model in an overall constricted healthy murine T-tubule. (**A**), Schematic representation of the morphology of the tubule (see **Table 1**). Membrane potentials (**B**), extracellular potentials (**C**), and simulated sodium current densities upon a voltage-clamp step of the tubule mouth from -85 to -20 mV (**D**) are given. The peak sodium current decreases in deeper segments, while activation is delayed and inactivation is faster in deeper segments of the tubules (**D**). This correlates with a lower driving force (**B**), and changes in the activation gates (m^3, **E**) and inactivation gates (*hj*, **F**). Peak open probability (defined as m^3hj) first increases, then decreases along the T-tubule (**G**).
